# What is a blink? Classifying and characterizing blinks in eye openness signals

**DOI:** 10.3758/s13428-023-02333-9

**Published:** 2024-02-29

**Authors:** Marcus Nyström, Richard Andersson, Diederick C. Niehorster, Roy S. Hessels, Ignace T. C. Hooge

**Affiliations:** 1 Lund University Humanities Lab, Box 201, SE–221 00 Lund, Sweden; 2 Tobii AB, Box 743, SE–182 17 Danderyd, Sweden; 3 Lund University Humanities Lab and Department of Psychology, Box 201, SE–221 00 Lund, Sweden; 4 Experimental Psychology, Helmholtz Institute, Utrecht University, Heidelberglaan 1, 3584 CS Utrecht, The Netherlands

**Keywords:** Blink, Eye tracking

## Abstract

Blinks, the closing and opening of the eyelids, are used in a wide array of fields where human function and behavior are studied. In data from video-based eye trackers, blink rate and duration are often estimated from the pupil-size signal. However, blinks and their parameters can be estimated only indirectly from this signal, since it does not explicitly contain information about the eyelid position. We ask whether blinks detected from an eye openness signal that estimates the distance between the eyelids (EO blinks) are comparable to blinks detected with a traditional algorithm using the pupil-size signal (PS blinks) and how robust blink detection is when data quality is low. In terms of rate, there was an almost-perfect overlap between EO and PS blink (*F*1 score: 0.98) when the head was in the center of the eye tracker’s tracking range where data quality was high and a high overlap (*F*1 score 0.94) when the head was at the edge of the tracking range where data quality was worse. When there was a difference in blink rate between EO and PS blinks, it was mainly due to data loss in the pupil-size signal. Blink durations were about 60 ms longer in EO blinks compared to PS blinks. Moreover, the dynamics of EO blinks was similar to results from previous literature. We conclude that the eye openness signal together with our proposed blink detection algorithm provides an advantageous method to detect and describe blinks in greater detail.

## Introduction

Blinks involve the closing and re-opening of the eyelids and have been the subject of investigations in a large variety of fields due to their relation with human behavior, function, and cognition (e.g., Stern et al., 1984; Eckstein et al., 2017). Traditionally, eyelid position has been estimated directly or indirectly with attachment devices such as electrooculography (EOG) or magnetic search coils (MSC), but it is increasingly being estimated from video recordings of participants’ faces. In data from many modern video-based eye trackers, blink classification is commonly performed on the pupil size (PS) signal, where clusters of samples reported as invalid (or data loss) are taken as blinks. However, the PS signal can have invalid samples for other reasons than eyelid closure, for instance due to head movements relative to the camera (Wass et al., 2014), and looking outside (Hessels et al., 2015a) or towards the edges of the screen (Nyström et al., 2013). Importantly, the eyelid position cannot be measured directly from the PS signal. Consequently, researchers need better methods to study different properties of blinks in more detail. In line with this need, some eye trackers output information about the position of the eyelids and/or information about how open the eye is, i.e., the distance between the upper and lower eyelids (e.g., the Smart Eye Pro, Tobii Pro Spectrum, and Tobii Pro Fusion). In this paper, we will investigate the following. First, we ask whether blink rate and blink duration differ when blinks are detected from the PS signal and a recently introduced eye openness (EO) signal from a commercial remote eye tracker (the Tobii Pro Spectrum).

Second, to investigate how robust blink detection from these signals is when tracking is difficult and data quality deteriorates, we evoke a situation where data loss and precision are expected to become worse. Besides recording blinks from participants where data loss is expected to be low and signal precision is expected to be high in the center of the head-box of the eye tracker, we also record blinks close to or far away from the eye tracker. This may happen, for example, when participants are free to move and end up at the edge of the eye tracker head-box. Finally, we test whether properties and dynamics of blinks detected from the eye-openness signal conform with previous literature.

Since it is advantageous to be able to detect blinks with a computer algorithm (Hooge et al., 2018), another contribution of this article is the development of a new algorithm for blink classification from the EO signal. To inform the design of this algorithm and identify key properties of blinks, we start by a systematic review of previous work of different approaches to define and describe, record, and classify blinks and compute their parameters. Informed by previous literature, the new algorithm for blink classification using the EO signal is proposed and its detected blinks are compared with blinks detected from commonly used algorithms operating on the PS signal (e.g., Hoogerbrugge et al., 2022). Data from 12 participants were collected and used in the comparison.

## Blinks

### Why are blinks interesting to study?

Blinks have been studied in several different fields of research and for different purposes (see overviews by e.g., Stern et al., 1984; Cruz et al., 2011; Eckstein et al., 2017). For instance, (Eckstein et al., 2017) discuss several ways blinks are informative of cognition and cognitive development, including how spontaneous blink rate is related to dopaminergic activity (Jongkees & Colzato, 2016), how blinks are associated with certain disorders and how different cognitive processes can be studied using blinks. Blinks have been recorded to study drowsiness, where Caffier et al. (2003) found that several parameters of spontaneous blinks are indicative of how drowsy a person is.

In particular, they found that blink duration, re-opening time, and closure duration changed reliably with the level of drowsiness. More generally, the percentage of how much the eyelids are closed (known as the PERCLOS measure) is often cited as the most valid and reliable indicator of sleepiness (Ahlström et al., 2021). In a vigilance task, McIntire et al. (2014) found that both blink rate and duration increased with the time-on-task as performance got worse. Blink rate has also been shown to increase when a person is being cross-examined in a courtroom (Ponder & Kennedy, 1927) and more generally to task demands (Stern & Skelly, 1984). In primates, Tada et al. (2013) found that the blink rate increased with the group size in which primates live and argue that blinks play a role in social communication.

When the eyelid is closed, less light reaches the retina. Despite the significant effect blinks have on the retinal image, the perceptual effects of blinks are typically small. Volkmann et al. (1980) showed that the sensitivity to visual stimulation on the retina is reduced before the eyelid starts closing and does not fully recover until after the eyelid is fully open (about 200 ms after blink onset). Blink suppression has a similar magnitude and time course as saccadic suppression (Matin, 1974; Volkmann et al., 1980).

### What is a blink and what types of blinks are there?

Humans are one of the many species that regularly open and close their eyelids. Blount (1927) provides four reasons why blinking is common in human and many non-human species. First, blinking is used to moisten the surface of the eyeball and to remove dirt from the eyes. Second, “to allow alteration to take place in the tension of the ocular muscles and thus to eliminate early fatigue” (p.120 Blount 1927). Third, to protect the eyes from injury due to external impact of the eyes, and finally, to protect the eyes against continuous exposure to light. In addition, (Cruz et al., 2011) write that blinks contribute to clarity of vision and to maintain the stability of tear film.

Similar to the reported confusion about terminology and definitions of fixation and saccades in the field of eye movement research (Hessels et al., 2018), there are surprisingly many papers about blinks that do not provide a clear definition of what a blink is. In those that do, blink definitions vary. In the remaining part of this subsection, examples of blink definition are provided together with a table that highlight such variations (Table 1).

**Table 1:** Example of different types of blinks and what aspects of them are mentioned when described in the literature

Type	Eyelid	Pupil	Cornea	Iris	Eyeball	Time	Space	Binocular	Ref
Blink	x	x			x	x		x	Blount (1927)
Wink	x	x			x	x			Blount (1927)
Half-blink	x								Blount (1927)
Flicker	x					x		x	Blount (1927)
Flink	x					x			Blount (1927)
Blink + full-closure of the eyes	x								Blount (1927)
Blink	x					x			Stern et al. (1984)
Small blink	x			x			x		Stevenson et al. (1986)
Medium blink	x	x					x		Stevenson et al. (1986)
Large blink	x						x		Stevenson et al. (1986)
Blink	x								Cruz et al. (2011)
Twitch	x						x		Patel et al. (2011)
Incomplete blink	x		x						Patel et al. (2011)
Complete blink	x		x						Patel et al. (2011)
Forced or voluntary blink	x								Patel et al. (2011)
Complete blink	x		x						Cardona et al. (2011)
Incomplete blink	x		x						Cardona et al. (2011)
Twitch blink	x		x						Cardona et al. (2011)
Twitch blink	x						x		Cruz et al. (2011)
Large blink	x		x				x		Rosenfield et al. (2015)

An ‘x’ means that a particular aspect of the blink is mentioned in the definition. In his description of blinks, for instance, Blount (1927) mentions that a blink is a temporary closure (time) of the eyelids (eyelid) in both eyes (binocular), where the pupil is momentarily hidden from view (pupil), but the eyeball does not necessarily move (eyeball)

Some of the earlier definitions of blinks were provided by Blount (1927), who distinguishes between eight different types of blinks, albeit admitting their rather arbitrary nature. Since the definitions also cover animals, not all types are applicable to humans; two involve the ‘third eyelid’ present in some animals. Therefore, only six are provided (definitions taken directly from the paper, pp. 111–112):

- *Blink* - is a temporary closure of both eyes, involving movements of the upper and lower eyelids. The pupil is momentarily hidden from view, but the eyeball does not necessarily move its position to an observable extent.
- A *wink* is a similar movement, but of one eye only. (It must not be confused with the human "wink," which is carried out voluntarily.)
- A *half-blink* may or may not involve the lower eyelids. The upper eyelids are always lowered and approach the lower lids.
- A *flicker* is a rapid, synchronous movement of both upper eyelids, but not a half-blink. Flickering is constituted of several flickers carried out in rapid succession.
- A *flink* is a flicker involving one eyelid only.
- A *full-closure of the eyes* is much the same as a prolonged blink, but the edges of the eyelids remain together for a considerable time.

More recently, (Cruz et al., 2011) defined a blink as an “eyelid movement that closes and opens the palpebral fissure”, where the latter refers to the opening between the eyelids. A similar definition was given by Stern et al. (1984) who write that an “Endogenous [spontaneous] eye blink is defined as fast closing and reopening of eyelids”. Stern et al. (1984) distinguish between three types of blinks: spontaneous, reflexive, and voluntary, where the two former are considered to be involuntary. Spontaneous blinks are characterized by “unconscious, transient, or brief closure of both upper eyelids that occurs in a highly symmetrical and coordinated fashion in the absence of any evident stimulus” (Cruz et al., 2011). Reflexive blinks occur in response to potentially injurious stimuli such as a large visual or auditory transient ones (Stern et al., 1984). Spontaneous and voluntary blinks cannot be distinguished objectively, without knowledge about the context in which they were recorded, according to Volkmann et al. (1980). Normally, the upper eyelid is responsible for most of the change in eye openness during a blink Cruz et al. (2011). However, Collins et al. (1989) write that during what they call a ‘forced’, voluntary blink, the lower eyelid moves upwards to meet the upper eyelid more than during spontaneous blinks, where the lower eyelid moves only slightly nasally.

(Patel et al., 2011 p. 201) provides a blink classification scheme including four types of blinks:

A twitch blink consisting of a small movement of the upper eyelid, an incomplete blink in which the descending upper eyelid covers less than two-thirds of the cornea, a complete blink in which the descending upper eyelid covers at least two-thirds of the cornea and a forced or voluntary blink in which the lower eyelid rises to meet the upper lid.

A slightly different scheme was used by  (Cardona et al., 2011 p. 193):

Blink rate included complete and incomplete blinks, in which a complete blink was defined by a downward movement of the upper eyelid covering more than 75% of the cornea. Minor twitches of the upper eyelid, covering less than 30% of the cornea, [...] Blinks covering between 30 and 75% of the cornea were counted as incomplete blinks.

Incomplete blinks have also been referred to as ‘microblinks’, which Stern et al. (1984) describe as endogenous blinks with reduced amplitudes. Cruz et al. (2011) refer to twitch blinks as a “small, almost undetectable movement of the upper eyelid”.

Stevenson et al. (1986) divided blinks into small, medium, and large. During small blinks, the upper eyelid typically descended to a position between the upper edge of the iris and the upper edge of the pupil. When most or all of the pupil was covered, a medium blink was registered. A large blink represented the case where the upper eyelid met with the lower eyelid.

Rosenfield et al. (2015) provided a similar definition where the requirement for a full blink was the “upper eyelid cover the entire corneal surface”.

Even though they are controlled by the same muscle (orbicularis oculi) (Sheedy et al., 2005), blinking has been qualitatively distinguished from squinting, where the latter narrows “the vertical dimension of the palpebral aperture to improve visual resolution while also lowering retinal illumination to minimize glare” (Rosenfield et al., 2015).

In everyday life, a combination of different types of blinks occurs. In a group of healthy participants, (Abelson & Holly, 1977) found that the majority (80%) of blinks were complete blinks where the eyelids closed completely; 17% were incomplete and 3% were twitch blinks. Carney and Hill (1982) found a percentage of complete and incomplete blinks of 90% and 8%, respectively, and Collins et al. (2006) reported 73% complete blinks, 22% incomplete blinks, and 5% twitch blinks.

Even though there are differences in how blinks are defined (e.g., does the pupil have to be covered by the eyelids or not, how much of the cornea has been be covered by the eyelids) and what types of blinks there are (e.g., spontaneous, forced, full, partial, and twitch blinks), a common denominator from the above literature seems to be that blinks are associated with a rather brief lid closure of one or both eyes (cf. Table 1). How much and how quickly the eyelids descend and re-open, how long they are closed, whether the blinks are monocular or binocular and what structure of the eye surface the eyelids need to cover have been used to decide the blink sub-type. Compared to other types of blinks, small movements of the upper eyelids (twitch blinks and flicker) seem to be mentioned only in a very small number of papers, leading us to question how relevant they are to the broader scientific community interested in blinks, for instance researchers interested in mental workload or arousal.

### Characteristics and kinematics of blinks

Blinks can be characterized by a number of parameters where the most common include the rate, duration, inter-blink interval, amplitude, closing time/speed, re-opening time/speed.

#### Blink rate and inter-blink intervals

The blink rate is perhaps the most used blink parameter. Blink rates and inter-blink interval durations in the literature vary substantially, both over participants, groups, and tasks. The blink rate within participants has been reported to be relatively constant, with a specific ‘rhythm’ or patterns of inter-blink intervals (Carney & Hill, 1982). Leigh and Zee (1999) [p. 156] write that blinks typically occur 20 times per minute and Carney and Hill (1982) report an average of rate of 12.55 blinks per minute over 20 healthy participants. Caffier et al. (2003) reported that blink rate decreases as participants become more drowsy (alert: 16.33 blinks per minute, drowsy: 15.84 blinks per minute). Blink rate changes over the course of life, where even fetuses blink (less than three times per minute) and the blink rate increases over childhood (6–8 blinks per minute) to stabilize at a rate or 20 blinks per minute during adulthood (Eckstein et al., 2017; Bacher & Smotherman, 2004).

Ponder and Kennedy (1927) reported inter-blink intervals in 31 subjects and found a left-skewed distribution with the majority of intervals occurring between 0.5 and 2 s and a minority of intervals above 5 s. However, some of the individual participants showed more Gaussian, rectangular, or bimodal distributions of inter-blink intervals.

Factors such as air quality, defensive responses, cognitive processes, situational demands, and individual differences influence the blink rate (Stern et al., 1984). Drew (1951) found that blink rate varies substantially across individuals, but that this inter-subject variation was not related to task performance. However, intra-subject variation in blink rate was correlated with the level of difficulty in tasks performed both in the laboratory and in a real-world driving task.

#### Blink duration and opening/closing times

According to Stern et al. (1984), most blinks last between 150 and 400 ms, where the eyes are completely closed for about 50 ms. Stern et al. (1984) also noted that the re-opening time was longer than the closing time. Caffier et al. (2003) report blink durations of about 200 ms in ‘alert’ participants, with a skewed blink waveform where the closing time (63 ms) was much shorter than the opening time (138 ms). Differences in duration between different types of blinks are also described in the literature. VanderWerf et al. (2003) found that blinks triggered by electrical stimulation of the supraorbital nerve triggered shorter and less variable blinks than spontaneous blinks, whereas blinks induced by air puffs were more similar to spontaneous blinks in terms of the same measures. Stern et al. (1984) found spontaneous eye blinks to be shorter than reflexive and voluntary blinks. Blink durations have been found to decrease with increasing visual task demands (Benedetto et al., 2011).

#### Blink amplitude

Blink amplitude refers to the distance between the eyelid position at a baseline value (e.g., the blink onset) to the maximum excursion from this baseline value during a blink (typically when the eye is maximally closed).

The eyelids do not necessarily return to the same position after a blink, thus the down- and the up-phase of the blink may have different amplitudes. According to Cruz et al. (2011), it is generally agreed that it is easier to estimate the amplitude of the down-phase, since the up-phase can show an oscillatory behavior and therefore it can be difficult to determine its offset. There is typically a large within-subject variability in blink amplitude. The main reason behind this variability is physiological; blinks starting during an upward gaze direction have longer amplitudes since the upper eyelid is pushed upwards prior to the blink (Stern et al., 1984). Conversely, blinks during a downward gaze are associated with shorter amplitudes.

Since blinks historically have been recorded with devices that output a voltage (Stern et al., 1984), the unit of blink amplitude is either given in $\mu$V (Tanaka & Yamaoka, 1993) or expressed in relative terms following a calibration. Helmchen et al. (2006) describe such a calibration procedure where the voltage at eyes open and eyes fully closed are recorded and subsequent values are expressed in relation to these. They propose the relative blink amplitude (RBA) where a 30% value, for instance, would mean 30% of full closure.

In video recordings of the eye, the position of the upper and lower eyelids can be measured frame-by-frame in millimeters, either manually or by means of computerized methods to estimate blink amplitude (Cruz et al., 2011). Recently, several methods using deep neural networks have been developed to estimate eye openness and blinks  (Cortacero et al., 2019).

Cruz et al. (2011) reported a relative blink amplitude, where the blink amplitude in millimeters was divided by marginal reflex distance (MRD), which is the distance between the upper eyelid and the pupil center. A relative blink amplitude above 1 means that the pupil is fully covered by the eyelid and Cruz et al. (2011) found in data from one participant recorded for 1 h that only 4.5% of the blinks do not fully cover the pupil.

Cruz et al. (2011) argue that a more natural unit to present blink amplitude would be in degrees, since the eyelid follows the curved surface of the cornea. Using magnetic search coils, the amplitude can be estimated as the angle of a coil taped/glued to the eyelid relative to the orientation of the magnetic field (Cruz et al., 2011). They report blink amplitudes ranging from 10 degrees to 60 degrees and also that blink amplitudes change with age; from 37.8 degrees ($SD = 4.6$) in 40–49-year-olds to 28.4 degrees ($SD = 2.5$) in a group of 80–89-year-olds (Sun et al., 1997)

#### Blinks and eye movements

Blinks both affect eye movements and are affected by eye movements. When fixating a stationary target, the eyes move down and towards the nose during a blink (pp. 156–157 Leigh and Zee, 1999), although such movements are slower than saccades. They also note that blinks frequently occur during saccades and that they become increasingly more common for larger saccades. Saccades during blinks are typically slower and have lower peak velocities and accelerations and longer durations (Rambold et al., 2002). Therefore, Leigh and Zee (1999) emphasize that blinks must be taken into account when studying saccades.

The eyelid position changes as a function of vertical eye orientation (Stern et al., 1984). Evinger et al. (1991) found that the kinematics of the eyelid during the opening phase of a blink is indistinguishable from eyelid kinematics when the eyelid moves due to a upward vertical saccade.

Recently, magnetic resonance imaging (MRI) was used to image the eyeball during blinks. Recording data from two participants, Kirchner et al. (2022) found that the eyeball can be lifted (inferior to superior displacement) and retracted (anterior to posterior displacement) with as much as 2 mm during a blink. For one of the participants, the eye also rotated vertically downward by 35 degrees.

## Methods to record blinks

Early methods to quantify blinks include visual observation as well as mechanical, optical, electric, and photoelectric methods. A good overview of these methods is given by Stern et al. (1984); Cruz et al. (2011); Eckstein et al. (2017). Over the past decades, three methods, electrooculography (EOG), magnetic search coils (MSC), and video recordings, are perhaps the most common.

EOG is commonly used to estimate eye movements and uses the fact that there is a potential difference between the cornea and the retina, generating an electric field around the eyeball. Consequently, any eye rotation moves this electrical field and electrodes around the eye can pick up resulting changes in voltage. Vertical eye rotation can be estimated by attaching electrodes above and below the eye, whereas horizontal eye rotation is estimated by attaching electrodes on the left and right side of the eye. The eyelid acts as a “sliding resistor” and changes the potential between the cornea and retina as it moves and the EOG signal can therefore be used to estimate eyelid movement and hence blinks (Stern et al., 1984). Since both the vertical orientation of the eye and the eyelid position contribute to EOG in the vertical plane, it is impossible to distinguish vertical eye movement from eyelid movement (Stern et al., 1984). While being a simple and inexpensive method to estimate eyelid position, EOG suffers from electrical and electromyographic noise, requires repeated calibration and has an unstable, drifting baseline value (Leigh & Zee, 1999).

The magnetic search coil technique is based on attaching coils to the eyelids and putting them in an alternating magnetic field (Remmel, 1984). An electric current is generated when the coils move within this magnetic field and this current can be related to the three dimensional spatial position and rotation of the coil. The magnetic search coil technique is known for being one of most accurate and precise methods to record eye movements (p. 723 Leigh and Zee, 1999) and eyelid movements (Guitton et al., 1991). The obvious disadvantage of the technique is its invasive nature, where coils need to be glued or taped to the eyelids (Evinger et al., 1991).

Several methods have been proposed to extract relevant features from a video recording of the eyes to be able to estimate blinks. For instance, Appel et al. (2016) and Espinosa et al. (2018) used the assumption that the (dark) pupil gets occluded by the eyelid during a blink, which makes frames acquired during a blink brighter than frames recorded when the pupil is visible. The summed pixels values in each frame therefore provide a crude estimate of the eyelid position.

Another common method to estimate blink parameters is to use eye landmark detection  (e.g., Soukupova and Cech, 2016; Cech and Soukupova, 2016), today implemented in open software such as OpenFace (Baltrusaitis et al., 2018) or MediaPipe (Lugaresi et al., 2019) and from these landmarks compute the eye aspect ratio (EAR), i.e., the ratio between the openness of the eye and its width (Alzahrani et al., 2021). Closed eyelids are thus represented with EARs close to zero. To detect blinks from the EAR, Soukupova and Cech (2016) used a linear SVM classifier trained on manually annotated data.

In many video-based eye trackers, the pupil area is estimated from the eye image. The resulting pupil-size signal is then used to estimate blinks, under the assumption that blinks occur when the samples in this signal are invalid due to eyelid closure. This type of blink detection is discussed in detail in “Blink classification in P-CR eye trackers”.

## Description and classification of blinks

Using the magnetic search coil technique, VanderWerf et al. (2003) considered the start of the down-phase of a blink to occur when the eyelid deviated more than 0.2^∘^ from a calibrated zero position (when the eye was fully open) and the end of the blink to occur when the up-phase amplitude of the blink reached 95% of the maximal down-phase amplitude (A_max_). The down-phase duration was defined as the time from the onset of the down-phase until the time A_max_ was reached. Consequently, the up-phase duration lasted from the occurrence of A_max_ to the blink end.

However, often much simpler operationalizations of blinks are provided. For instance, using EOG data, Barbato et al. (2000) operationalize a blink “as a sharp high amplitude wave $\ge$100 $\mu$V and <400 ms in duration.” Often, human pattern recognition is used to identify blinks from a recorded signal. For instance, Evinger et al. (1991) write that:

the investigator identified the maximum down-phase (lid closing) velocity, the maximum up-phase (lid opening) velocity, the beginning of the blink or lid saccade, the maximum downward lid excursion and the end of the blink or lid saccade

### Blink classification in P-CR eye trackers

**Fig. 1:**
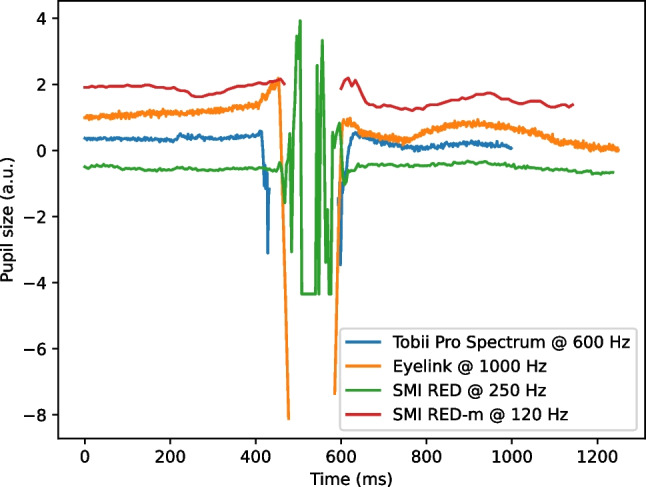
Blinks as they typically appear in the pupil size signal from four different P-CR eye trackers. For an eye tracker with a high sampling frequency (e.g., the EyeLink), the blink is preceded/succeeded by a quick decrease/increase in the pupil size signal, reflecting the gradual occlusion of the pupil in the eye image (cf. Fig. 2). For an eye tracker with a lower sampling frequency (e.g., the SMI RED-m), this gradual occlusion is typically not visible

**Fig. 2:**
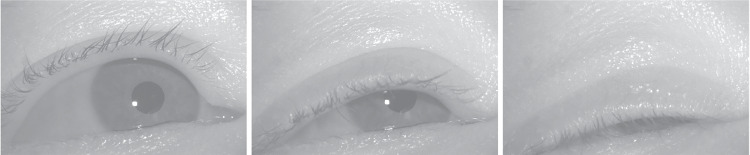
Eyelid position at different stages of a spontaneous blink. Note that when the upper eyelid starts covering the pupil (*middle*), the center-of-mass of the visible part of the pupil is lower compared to when the eye is fully open (*left*) and the pupil size value in the PS signal becomes smaller (cf. Figure 1). Eye images were recorded with the FLEX setup (Hooge et al., 2021; Nyström et al., 2022)

Pupil and corneal-reflection (P-CR) eye trackers use the locations of the pupil and one or several corneal reflections in an image of the eye to estimate the gaze direction. In P-CR eye trackers, a blink is characterized by data loss, since the eyelid covers both the pupil and the corneal reflection as the eyelid is fully closed. Depending on the sampling frequency of the eye tracker and the specific implementation of the pupil center localization algorithm, this period of data loss can be preceded and followed by a quick change in estimated pupil size (see e.g., the EyeLink signal in Fig. 1), reflecting the gradual occlusion of the pupil (and hence change in its estimated center-of-mass) when the eyelid closes and re-opens (Fig. 2).

Many of the papers reporting blink measures briefly describe the procedure for detecting blinks in the method section and use either the pupil signal alone or a combination of the pupil and the gaze signal together with a minimum blink duration (p. 177 Holmqvist et al., 2011). The approaches range from simply regarding periods where data are lost as blinks (Brouwer et al., 2005) to considering several criteria such as pupil size and pupil dilation velocity, as well as the position of gaze on the screen  (gaze position data outside of the screen may be indicative of blinks, for instance Karatekin et al. 2007). Recent examples of simple data loss criteria to detect blinks are given by  Hoogerbrugge et al. (2022), who used periods where “no pupil data was measured”, which were longer than 30 ms and shorter than 3 s and Demiral et al. (2022) considered intervals between 100 and 400 ms where the pupil diameter signal was missing or smaller than 25 units as blinks.

The paper by Pedrotti et al. (2011) is one of the first papers that systematically investigates blink detection in data from a P-CR eye tracker. Using the pupil diameter signal at 50 Hz from an SMI RED (Sensomotoric Instruments, Teltow, Germany), they devised an algorithm consisting of two steps: preprocessing and blink detection. In the pre-processing stage, values in the pupil and gaze signals indicative of a closed or partially closed eyelid were set to zero. This included samples recorded as invalid by the eye tracker, outliers in pupil diameter, and samples with low values in the horizontal or vertical gaze signals ($(x + y) < 10$ pixels). The latter condition reflected artifacts in the gaze signal that provided small, sometimes negative values in the gaze signal due to the blinks. Periods of zeros in the preprocessed signal were considered indicative of blinks, which were defined to start 60 ms before the first zero and end at the last zero in the blink period. The 60 ms were motivated by visual observation of the video image of the eye, which revealed that the eyelid started to descend 60 ms before the first zero in the blink period. In comparison to visual inspection of concurrently recorded eye videos, Pedrotti et al. (2011) found that 97% of the blinks were classified correctly. However, only 79% of the blink durations were estimated correctly, where a correct duration could deviate with ±40 ms at both the onset and offset.

Mathôt (2013) proposed a blink detection method to allow subsequent reconstruction of pupil size (PS) signals corrupted by blinks. In data from an EyeLink 1000, he used the fact that the PS signal changes quickly on each side of a blink and identified blink onsets and offsets using the pupil size velocity signal (cf. Fig. 1). A blink was considered to start when the PS velocity exceeded a threshold of five arbitrary units. The offset was reached when the velocity went back to zero. Four parameters need to be set by the experimenter. First, the PS signal was low-pass filtered before velocity calculation, which required the length of the filter to be set. Additional parameters included velocity thresholds at onset and offset and a short addition (Mathôt used 10 ms) to the estimated blink duration (offset - onset), since visual inspection of eye videos revealed that his approach generally underestimated the blink duration with this amount.

A similar method was proposed by Hershman et al. (2018), who implemented a blink detection algorithm that first identified all periods of data loss and then searched for the onset/offset of a blink starting from the onset/offset of the data loss period. The blink onset was located by first low-pass filtering the PS signal and then moving backwards from the data loss onset until the filtered signal no longer was monotonically increasing. This point, the authors argue, reflects the “end of the eyelid signal and the start of the measurement noise”. Blink offsets were identified in the same manner, by moving forward in time from the location of the data loss offset.

Partial blinks do not necessarily lead to data loss in P-CR eye trackers, since the pupil may still be (partly) visible in the eye image (see middle image in Fig. 2). Brych et al. (2021) did not rely on periods of data loss to detect blinks, but instead looked at how much the pupil diameter deviated from the average pupil diameter; a blink was detected if the z-transformed pupil diameter went below two standard deviations of the mean. Moreover, blinks shorter than 50 ms or longer than 500 ms were discarded. Those that were too close in time (100 ms) were merged into one.

A similar approach was employed by Coe et al. (2022). However, they found it problematic to detect blinks using deviations from the mean pupil diameter due the large variation in pupil size over time. Therefore, they de-trended the pupil diameter signal prior to applying fixed thresholds for blink detection.

Some authors argue that physiologically unlikely pupil size values should be excluded prior to further processing of the pupil size signal. For examples, Kret and Sjak-Shie (2019) removed all non-positive pupil size values and Alnæs et al. (2014) excluded pupil values with a diameter smaller than 2 mm or larger than 7 mm and also 50 ms on each side of the gap such exclusions caused.

## Proposed algorithm for blink classification using eye openness signals

From e.g., Pedrotti et al. (2011), it is clear that the occurrence of blinks where the eyelids mostly or fully occlude the pupil can accurately be detected with the P-CR eye tracker they used. However, durations were more difficult to estimate accurately, which is unsurprising since no direct information about the eyelids was available from the PS signal. For the same reason, blink parameters such as blink amplitude and eyelid velocity were not possible to estimate at all solely based on a PS signal. Consequently, for more detailed research on blinks and their parameters, there is a need for methods that estimate the eyelid position or eye opening directly.

**Fig. 3:**
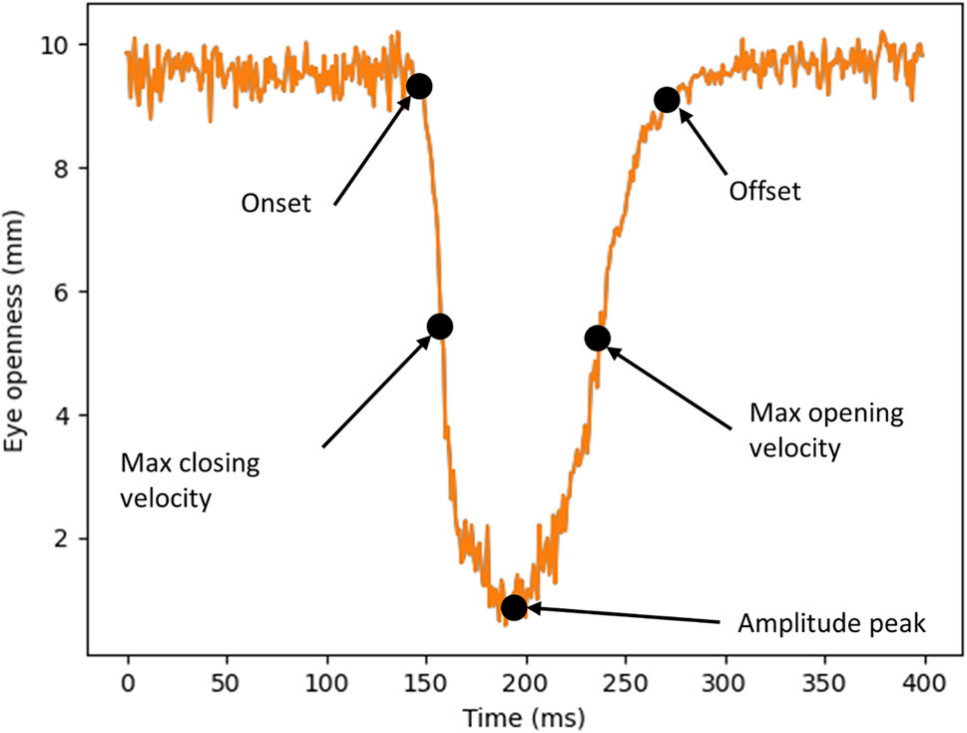
Waveform of a blink recorded with the Tobii Pro Spectrum at 600 Hz. Key locations detected with the proposed algorithm and used to derive blink measures, are indicated in the figure

We propose an algorithm to detect blinks and their parameters using the EO signal from a commercial eye tracker (Tobii Pro Spectrum), where eye openness is defined as (with respect to the eye image) “the diameter in millimeters of the largest sphere that can be fitted between the upper and lower eyelids”^1^. Thus, it provides a continuous measure of the distance between the upper and lower eyelids. Figure 3 illustrates a blink waveform recorded from a participant using this eye tracker.

What can we learn from the above literature review about blinks and how does it help us to make design choices when developing a new blink classification algorithm? The general consensus is that a blink is a brief eyelid closure followed by a brief eyelid opening, can vary in amplitude, and can be both monocular and binocular. As such, the eyelid opening transitions smoothly from a large value to a smaller value and then back again to a larger value, forming a smooth peak-like waveform when plotted over time as can be seen in Fig. 3. The goal of the algorithm is thus to classify everything that resembles this typical waveform, i.e., a high-velocity eyelid closure followed by high-velocity eyelid opening. The resting period between opening and closing stages can be short (as in the case of a spontaneous blink) or long (in case of a voluntary, prolonged eyelid closure). The default parameters of the algorithm are informed by previous literature, although we have made a conscious decision to exclude very small amplitude blinks, e.g., microblinks (Stern et al., 1984) and twitch blinks (Cruz et al., 2011) from being detected, since they are rarely mentioned in the literature. That said, the algorithm can easily be tweaked to the needs of researchers who are interested in such blinks. Finally, we cannot use the relationship between the eyelid position and other features of the eye such as the pupil or sclera to classify blinks into different subtypes (cf. Table 1, since we are using an EO signal from an eye tracker that does not provide the required information.

**Table 2:** Settings used to detect blinks from the eye openness signal

Setting	Value	Comment
Gap fill	40 ms	interpolate missing data in intervals < 40 ms
Filter length	25 ms	Length of Savitzky–Golay filter
Min blink amplitude	10% of fully open	*cf.* table caption.
Min peak opening/closing vel.	$2 \cdot \text {MAD}(v)$	The lowest one need to exceed $2 \cdot \text {MAD}(v)$
Min blink duration	30 ms	
Blink merge	100 ms	Merge blinks closer in time than 100 ms

The median value of the eye openness signal represents when the eyelids are considered to be ‘fully open’

The blink detection algorithm is implemented as follows (settings used in the algorithm are summarized in Table 2):

1. Interpolate segments of data loss in the EO signal if the segment is shorter than 40 ms. A similar maximum duration threshold is used by the I-2MC algorithm for fixation classification (Hessels et al., 2017).
2. Low-pass filter the EO signal with a Savitzky–Golay filter of size 25 ms using a second-order polynomial.
3. Find peaks in the EO signal (a similar strategy was employed by e.g., Caffier et al., 2003), using the find_peaks function from the Python scipy package (v. 1.10.0).
4. Compute the velocity *v* of the (un-filtered) EO signal as the first derivative of the EO signal using a second order Savitzky–Golay filter of length 25 ms.
5. For each peak, go backward/forward in time from the peak center until *v* is below a threshold $T_{vel} = 3 \cdot \text {MAD}(v)$, where MAD denotes the median absolute deviation. These operations define the onset and offset of a blink candidate. Similar strategies have been used for e.g., saccade detection (Nyström & Holmqvist, 2010) and blink detection (Hershman et al., 2018).
6. Find the maximum velocity between the onset and the peak (peak opening velocity) and between the peak and the offset (peak closing velocity).
7. Reject blink candidates with too low peak opening or peak closing velocity, too short amplitude or duration according to Table 2.
8. Merge blinks separated by less than 100 ms (Brych et al., 2021).

For each blink, the algorithm outputs its onset, offset, eye openness at onset/offset/amplitude peak, time of amplitude peak, maximum closing/opening velocity, time when maximum closing/opening velocity is reached. For convenience, also the duration, opening and closing amplitudes are given, even though they can be computed from the previous measures. Some of these properties are illustrated in Fig. 3.

The algorithm was implemented with Python 3.10. Source code along with example usage and data are available from https://github.com/marcus-nystrom/BlinkDetector.

## Data collection

Eye openness and pupil data were collected to investigate whether blinks detected from the PS signal differ from blinks detected from the EO signal. To test how robust blink detection from these signals were under non-optimal head positioning, we further recorded data while participants were positioned at three different locations in the eye-tracker headbox, i.e., at three different eye tracker-participant distances perpendicular to the screen: in the center of the headbox, at the edge of the headbox close to the eye tracker, and at the edge of the headbox far away from the eye trackers. In the following, these recordings will be referred to as center, near, and far. This study was approved by the Swedish Ethical Review Authority (Dnr 2019-01081).

### Participants

We recorded data from 12 participants (eight males and four females; age: $M = 50.2$, $SD = 6.4$ years), who were employees at Lund University, Lund, Sweden.

### Materials

Data were collected in the Digital Classroom at the Lund University Humanities Lab, Lund, Sweden, that is equipped with 16 identical Tobii Pro Spectrum eye trackers (firmware v. 2.6.1). General room illumination came from overhead fluorescent lighting. Binocular eye movement data were collected with the Titta Toolbox v. 1.0 (Niehorster et al., 2020), using the Python SDK (v. 1.10.2) from Tobii.

Stimuli were shown on the native Tobii Pro Spectrum screen (EIZO FlexScan EV2451) with a resolution of 1920$\times$1080 pixels (52.8$\times$29.7 cm) and presented with PsychoPy (v. 3.1.2). The stimuli consisted of a black-and-white fixation target known to elicit stable fixations (Thaler et al., 2013 ABC in the lower panel of their Fig. 1) with a diameter of 0.5 degrees presented on a mid-gray background (gray value 128).

### Procedure

Each participant was seated in front of one of the Tobii Pro Spectrums in the Digital Classroom. They were calibrated and validated with the default 5-point calibration and 4-point validation in the Titta toolbox (Niehorster et al., 2020). Following the calibration procedure, they were asked to place their eyes in the center of the eye tracker headbox and blink every 1 second, prompted by a change in color of the fixation target (black was changed to white and vice versa). The same task was repeated while placing the eyes close to the edge of the headbox, both close to or far away from the eye tracker. The edges of the head box were operationalized as the eye positions relative to the eye tracker where the head circle visualization in the Titta toolbox started to flicker, indicating unstable tracking of the pupil and/or the CRs by the Tobii Pro Spectrum. On average, this occurred when they came closer than 51.4 cm ($SD=3.2$), or further away than 81.6 ($SD=3.7$) cm, from the eye tracker. No chinrest was used.

Each of the three conditions (center of headbox, close to eye tracker, far away from eye tracker) lasted for 60 s and came in a fixed order (center, near, and far). All participants were recorded in the same room at the same time.

**Table 3:** The percentage of data loss in the pupil size (PS) and eye openness (EO) signals

Condition	Data loss		RMS-S2S precision	
	PS (%)	EO (%)	PS (mm)	EO (mm)
Center	16.9	0.76	0.004	0.200
Near	23.7 (1.4x)	0.82 (1.1x)	0.013 (3.0x)	0.147 (0.7x)
Far	33.9 (2.0x)	1.13 (1.5x)	0.052 (12.2x)	0.248 (1.2x)

Participants were sitting in the center of the headbox (center), close to the eye tracker (near), and far from the eye tracker (far). The numbers is parenthesis represent the proportional change in value from the center condition (data loss in the PS signal is 1.4 times larger in the near condition compared to the center condition, for instance)

### Data analysis

Our proposed blink algorithm is used to detect blinks in the EO signal. For PS blink detection, we implement a ‘standard’ algorithm operating on the PS signal using the following steps:

1. Interpolate segments of data loss in the PS signal if the segment is shorter than 40 ms. A similar maximum duration threshold is used by the I-2MC algorithm for fixation classification (Hessels et al., 2017).
2. Identify the onsets and offsets of the remaining segments of data loss (cf. e.g., Hoogerbrugge et al. 2022).
3. Merge segments separated by less than 100 ms (Brych et al., 2021).

Note that steps 1. and 3. are used also when detecting EO blinks.

## Results

We asked how similar blinks are when computed from the pupil size signal (PS blinks) and the eye openness signal (EO blinks) in terms of rate and duration and how robust these similarities are to reductions in data quality—manipulated by changing the position of the head relative to the eye tracker. To gain insight into why EO and PS blinks sometimes differ, we investigate the relationship between data quality and these differences. Finally, we asked whether the systematic relationship between amplitude and peak velocity of the closing/opening phases of blinks observed in previous studies (e.g., Evinger et al. 1991) is also observed in EO blinks.

Following the recommendation in Dunn et al. (2023), we begin by providing information about the quality of the eye-tracking signals used (PS and EO signals) in terms of precision and data loss.

**Fig. 4:**
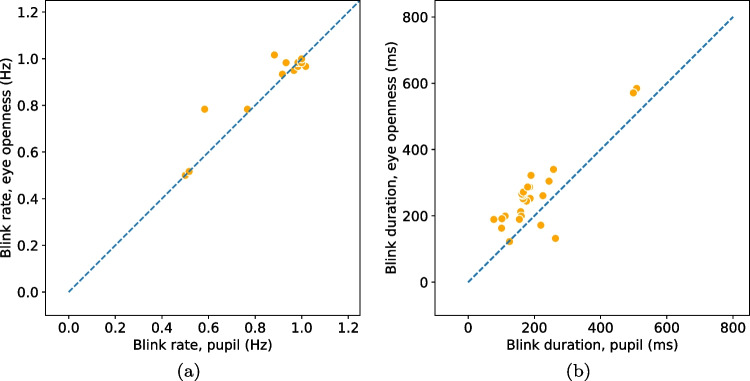
Blink rate (**a**) and duration (**b**) detected from the eye openness and pupil size signals when participants are located in the center of the head box. Each marker represents the average blink rate/duration per participant and eye. Markers that lay on the unity line (*dashed*) indicate that a similar number of blinks (*rate*) or similar durations were found in blinks from both signals

### Quality of the eye-tracker data

Due to temporary shutdowns of the eye tracker illumination in three of the eye trackers, three of the 36 trials (12 participants did three trials each) were missing 15.0%, 9.6%, and 4.2% of the samples (they were unexpectedly not reported by the eye tracker). Since the data quality in these trials was otherwise high, we did not exclude them from further analysis. However, the unexpectedly missing data were not taken into account when reporting the percentage of data loss, which was operationalized as the number of invalid samples reported by the eye tracker divided by all samples reported by the eye tracker.

Table 3 lists the percentage of data loss and RMS-S2S precision in both PS and EO signals across all participants. RMS-S2S was computed as the root mean square of distances between consecutive samples in the signals. While 17–34% of the data were reported as lost in the PS signal, there was negligible sample loss in the EO signal (<1.1%). Note that part of this difference was expected since eye openness, but not pupil size, can be estimated when the eyelids are closed. The percentage of lost data was the lowest when participants were positioned in the center of the headbox (center) and became larger as the distance from the center increased. Most samples were lost when participants were positioned far away from the eye tracker.

Precision in the PS signal followed the same general trend as data loss (best in center, followed by near, and then far). However, precision of the EO signal becomes higher (better) when moving closer to the camera (near) compared to the center position (center). Precision in the center condition was still higher than in the far condition.

Importantly, the relative differences between conditions for both data loss and precision were smaller in the EO signal compared to the PS signal, indicating that data quality in the EO signal was less influenced by the participant’s position in the head box (cf. values in parenthesis in Table 3).

**Fig. 5:**
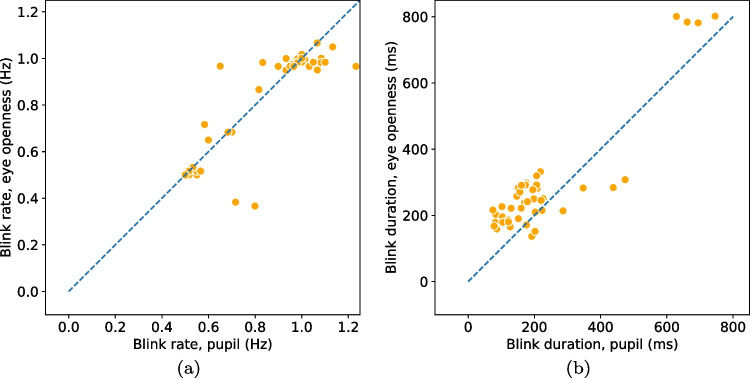
Blink rate (**a**) and duration (**b**) detected from the eye openness and pupil size signals when the head was positioned at the edge of the headbox (near or far from the eye tracker). Each marker represents the average blink rate/duration per participant and eye. Markers that lay on the unity line (*dashed*) indicate that a similar number of blinks (*rate*) or similar durations were found in blinks from both signals. Note that the number of markers is twice as many as in Fig. 4 since markers include data from two conditions (near and far), whereas data in Fig. 4 include data from only one condition (center)

### Do PS blinks and EO blinks differ in terms of rate and duration?

**Fig. 6:**
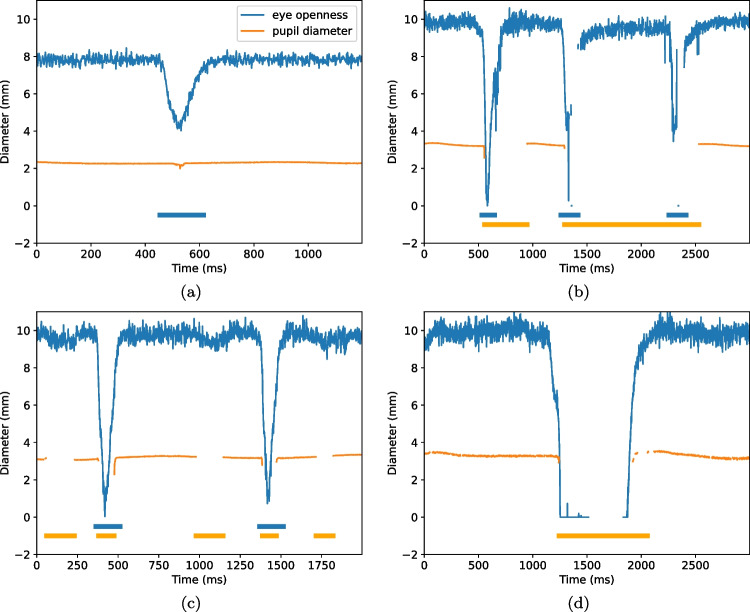
Examples of situations where the blink durations differ in the pupil size and the eye openness signals. The *y*-axis denotes the diameter of the pupil and the eye openness (the diameter of the largest circle that fits between the upper and lower eyelids). *Thick orange lines* at the bottom of the plot indicate segments where blinks were detected in the pupil size signal and *blue lines* where blinks were detected in the eye openness signal

Since we ask participants to perform a simple task—to blink at a rate of 1 Hz—we expect the two algorithms to detect one blink per second in their respective signals. In Fig. 4(a), blink rate in the PS signal is plotted against blink rate in the EO signal. If the two algorithms deliver a similar number of blinks, we expect all data points to lie on the unity line (dashes). As can been seen, there were only small differences in rate between EO blinks ($M = 0.88$ Hz, $SD = 0.18$ Hz) and PS blinks ($M = 0.87$ Hz, $SD = 0.18$ Hz) when participants were positioned in the center of the eye-tracker’s headbox. Only for two participants, the difference was more than three blinks. In these cases, incomplete, low amplitude blinks were detected in the EO signal but did not influence the PS signal enough to trigger a detection. An example of this situation is shown in Fig. 6(a).

Despite blink rates being similar, it could be that completely different blinks are detected in the two signals. One way to investigate this is to check whether the blinks from the two signals overlap in time. To quantify this type of overlap, we first match blinks according to the method described in Hooge et al. (2018) and then compute the event-based *F*1 score, where 0 indicates a low similarity and 1 a high similarity. Blinks in the center condition had an almost perfect match (*F*1 score: $M=0.98, SD=0.03$).

Blink durations, shown in Fig. 4(b), were 62.1 ms longer for EO blinks ($M = 261.5$ ms, $SD = 112.9$ ms) compared to PS blinks ($M = 199.4$ ms, $SD = 105.2$ ms). This is evident from the figure as the majority of data points are located above the dashed line. However, for two of the participants in the center condition—the two orange dots below the line in Fig. 4(b)—PS blinks were longer than EO blinks. Figure 6(b) shows an excerpt of data from one of these participants, where data loss in the PS signal creates very long blinks when the EO signal reveals that the eye is in fact open.

Blinks detected from the EO signal in the center condition started on average 32.8 ms ($SD=12.9$) earlier and ended 30.2 ms ($SD=56.3$) later compared to PS blinks.

### How does a reduction in data quality influence the similarity between EO and PS blinks?

To evaluate how a reduction in data quality influences the difference in rate and duration of EO and PS blinks, participants were asked to perform blinks while positioning their heads close (near) or far away (far) from the eye tracker, at the edges of the head box of the eye tracker. As already discussed, changing the position of the head away from the center of the headbox led to the expected reduction in data quality for both the near and far condition (Table 3). Since we are interested in how reduced data quality—rather than the position of the head in the headbox—influences blink detection, data from the near and far conditions were combined into a what we will henceforth refer to as the edge condition.

The absolute difference in blink rate (*R*) between EO and PS blinks was higher in the edge condition ($\bar{R}_{\text {EO}} - \bar{R}_{\text {PS}} = -0.05$ Hz) compared to the center condition (0.01 Hz). In the edge condition, the rate of PS blinks ($M = 0.90$ Hz, $SD = 0.21$ Hz) was higher than the EO blink rate ($M = 0.85$ Hz, $SD = 0.21$ Hz) (Fig. 5(a)). Figure 6(c) shows an example of data from the edge condition where three PS blinks were detected in the PS signal but not in the EO signal. Note that the EO signal is without any data loss and shows no indication of any blink, but the PS-based algorithm reacts to the gaps in the PS signal and identifies blinks. An example where a PS blink, but not an EO blink, is detected is given in Fig. 6(d). Here, the EO blink seems to be present in the data, but no EO blink is detected since the period of data loss just before the opening period is too long to be repaired by interpolation and thus no peak is found by the peak-detection algorithm.

The overlap between EO and PS blinks in the edge condition was high (*F*1 score, edge: $M=0.94, SD=0.10$), but lower than when participants were located in the center of the headbox (*F*1 score, $M=0.98, SD=0.03$). What aspect of data quality was responsible for this decrease in overlap in the edge condition? To address this question we calculated the Pearson correlation between the similarity (*F*1 scores) and two aspects of data quality (data loss and precision) for both signals. The only significant correlation ($p \le 0.05$) was found between similarity and data loss in the PS signal ($r = -0.48, p < 0.001$), indicating that the more data that were lost in the PS signal, the less similar were EO blinks and PS blinks. The other correlations were non-significant: EO signal/data loss: $r = -0.21, p = 0.08$; EO signal/precision: $r = 0.01, p = 0.96$; PS signal/precision: $r = -0.14, p = 0.23$.

Durations of blinks in the edge condition (EO blinks: $M = 281.5$ ms, $SD = 163.1$ ms; PS blinks: $M = 222.3$ ms, $SD = 161.4$ ms) were longer than those in the center condition by about 20 ms. However, the difference in duration (*D*) between EO blinks and PS blinks did not change much due to the reduction in data quality (Center: $\bar{D}_{\text {EO}} - \bar{D}_{\text {PS}} = 62.0$ ms; edge: $\bar{D}_{\text {EO}} - \bar{D}_{\text {PS}} = 59.2$ ms).

### Blink properties and dynamics

Having access to the EO signal enables blink measures other than rate and duration to be computed which is not possible when blinks are detected from the PS signal. Besides eye openness itself, this includes the amplitude, duration, and peak velocity of the opening and closing phases of a blink. The eye openness before a blink ($M = 8.75$ mm, $SD = 1.56$ mm) was generally larger than after a blink was completed ($M = 8.16$ mm, $SD = 1.67$ mm). The eyes were on average almost fully closed when the eye openness was at its minimum during a blink ($M = 0.73$ mm, $SD = 1.29$ mm).

**Fig. 7:**
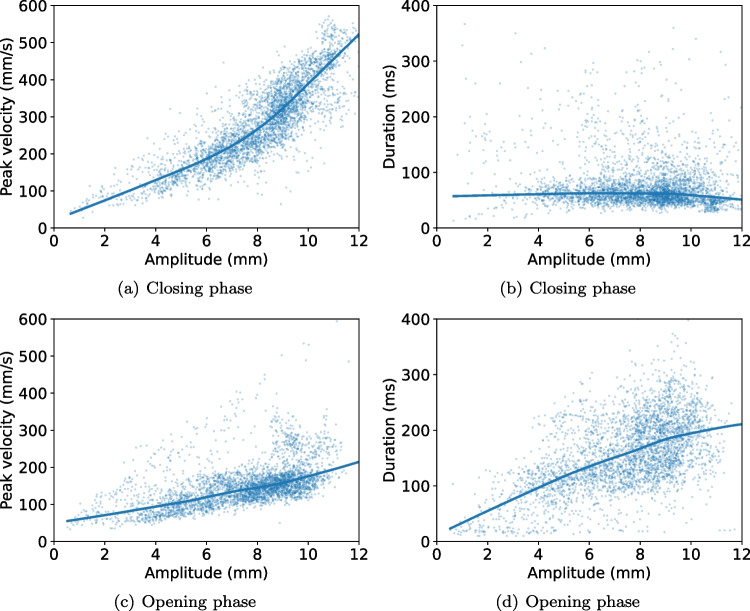
The relationship between amplitude, duration, and peak-velocity of blinks for the eyelid closing and eyelid opening phases. Each *do*t represents one blink. Data from all participants and conditions are shown. *Lines* represent locally weighted linear regression fit using regplot in seaborn (v. 0.12.2)

Figure 7 shows the relationship between the amplitude and peak velocity of blinks, as well as the blink amplitude and duration, separated for the eyelid closing and opening phases. Amplitudes ($M = 8.09$ mm, $SD = 2.01$ mm) and peak velocities ($M = 285.4$ deg/s, $SD = 104.5$ deg/s) are higher during the closing phase compared to the opening phase ($M = 7.50$ mm, $SD = 2.12$ mm; M = 151.4 deg/s, $SD = 59.1$ deg/s).

## Discussion

We asked whether it matters if blinks are detected from the pupil size (PS) signal or a newly developed eye openness (EO) signal from a commercial remote eye tracker, in terms of how many blinks are detected and what their properties are. We were also interested in how robust blink detection from these signals was under less optimal data quality and whether dynamic properties of blinks detected in the EO signal conformed with previous literature. Based on a comprehensive review of previous literature on blink definitions, dynamics, and classification, a new algorithm for blink detection using the EO signal was developed.

Traditionally, blinks have been detected from the PS signal (e.g., Hoogerbrugge et al., 2022; Demiral et al., 2022). When sitting in the center of the headbox, we found that the number of blinks detected from the PS signal (PS blinks) was similar to the number of blinks detected from the EO signal (EO blinks). Consequently, it does not matter which signal researchers use for blink detection when rate is the main outcome measure. Even when participants were located at the edges of the head-box, where tracking is expected to be worse, the similarity between PS blinks and EO blinks remained high (*F*1 score of 0.94, compared to 0.98 in the center of the headbox). It is worth noting that the higher amount of data loss in the PS signal at the edges of the head box (cf. Table 3) naturally leads to worse blink detection; if there are no data or significant data loss, blinks that actually occur become undetected or only partially detected, whereas data loss in the PS signal may also be misinterpreted as blinks. In fact, there was a significant Pearson correlation between the amount of data loss in the PS signal and the similarly between PS and EO blinks ($r = -0.53, p < 0.001$), indicating that the more data that were lost in the PS signal, the less similar were EO blinks and PS blinks.

Blinks detected from the EO signal were systematically longer than PS blinks, by 30 to 90 ms, and EO blinks both started earlier and ended later. This is unsurprising since the eyelids can begin to close at the onset of a blink and re-open during the termination of a blink, without covering any part of the pupil. Such eyelid movements affect the EO signal but not the PS signal. In the extreme case, an entire partial blink may occur, with no or minimal influence on the PS signal (cf. Fig. 6)(a), which means that no blink is detected in the PS signal.

For blinks that co-occur in both signals, can a constant value simply be added to PS blink durations such that they equal those of EO blinks? (Pedrotti et al., 2011) considered blinks to start 60 ms before samples were reported as invalid due to the eyelid covering the pupil in the eye image. This was based on visual observation of the eye videos revealing that the eyelid started to descend about 60 ms before the period of data loss. However, they found that, despite increasing the duration of all blinks with 60 ms, only 79% of the blink durations were estimated correctly despite a rather generous definition of what a ‘correct’ duration was ($\pm 40$ ms at both onset and offset). In agreement with Pedrotti et al. (2011)’s data, we found that adding a fixed value to PS blink durations would only partially make them approach EO blink durations, since there was a high variability in when PS and EO blinks started and, in particular, ended. In contrast to Pedrotti et al. (2011)’s observations from the eye video, we observed a 30-ms difference at both the onset and the offset of a blink when comparing PS and EO blinks and not only a 60-ms difference at the onset.

We have shown that it does not matter whether PS blink or eye blinks are used if rate is the main outcome measure and data quality is high. In the case of blink duration, onsets and offsets of EO blinks can be estimated with higher accuracy since the distance between the upper and lower eyelids is explicitly available. Moreover, the EO signal is more robust to less-optimal head positioning in the head box, making it the preferred choice when behavior resulting in such positioning is expected from the experimental task or participant group. An obvious advantage of using the EO signal is the ability to go beyond basic measures such as rate and duration. We could replicate some aspects of blink dynamics from previous work.

In line with previous literature on blinks (*cf.* their Fig. 2 Evinger et al., 1991), the closing phase of the blink generally had a higher peak velocity and a shorter duration than the opening phase. However, unlike (Evinger et al., 1991), we did not find a linear relationship between amplitude and peak velocity (Fig. 7). It should be noted that Evinger et al. (1991) used data from both voluntary blinks, spontaneous blinks, and reflex blinks, while we included mainly voluntary blinks. It is unclear if this is the source of the differences, in particular since Evinger et al. (1991) write that the blinks they recorded had similar waveforms irrespective of their origin (reflexive, spontaneous, voluntary). Importantly, other studies that reported a linear relationship between amplitude and peak velocity estimated amplitude in degrees from data recorded with magnetic search coils (Evinger et al., 1991; Garcia et al., 2013) and not mm from a video-based system as in this study (cf. “Blink amplitude”). This is, however, unlikely a source for the observed difference since the relationship between degrees and mm is approximately linear for amplitudes within the range of typical blinks ($\pm 30$ degrees which corresponds to a range about 12 mm).

To be able to compare EO and PS blinks, we designed a new EO blink detection algorithm informed by previous literature. We used the fact that blinks form a distinct and smooth peak-like waveform over time (see Fig. 3). Therefore, a peak detection method was at the heart of the EO blink algorithm. However, we noticed an issue with this design choice: data loss in the EO signal seems to be overrepresented during the opening phase of a blink in data from the particular eye tracker we tested (cf. Fig. 6(d)). When this period of data loss is too long to repair through interpolation, this leads to the situation that no peak—and therefore no blink—is detected. The origin of such data loss is unknown to us and we do not know if this is representative for other methods estimating eye openness from a video image of the eye.

An alternative to peak detection would be to identify blinks directly from the velocity of the EO signal, using a threshold similar to what has been done for saccade detection (Engbert & Kliegl, 2003; Smeets & Hooge, 2003; Nyström & Holmqvist, 2010). For spontaneous blinks that typically have high velocities and amplitudes, this would likely provide an accurate method, in particular for identifying the closing phase with its high velocity and short durations. However, it would be difficult to detect small and slow blinks that, despite showing the typical blink waveform, would have velocities that would be too low for a threshold-based method to handle robustly.

Our work could be extended in several ways. First, we have tested static head and eye positions in the center and at the edges of the headbox. Consequently, we do not know how data quality of the PS and EO signals would be affected by head or eye movement within the headbox. This could be a relevant investigation for certain target groups like infants, where significant head movement is expected both at the edges and within the headbox (Hessels et al., 2015a, b; Niehorster et al., 2018). Also, from e.g.,  Evinger et al. (1991), we know that eye movements complicate the detection of blinks, since changes in eye openness due to a blink and a vertical saccade may be indistinguishable. Future versions of a blink algorithm could be extended to separate between the contexts of such eyelid movements. Second, we have tested the blink detection algorithm at 600 Hz, without conducting a systematic comparison of whether similar differences between EO and PS blinks would occur at other sampling frequencies. Moreover, a possible extension of the EO blink detection would be to further classify blinks into subclasses such as full, partial, or micro-blink. However, since the literature does not seem to converge on these definitions, we have chosen to provide the user with comprehensive properties of each blink and leave it to the user to design sub-classification schemes that are suitable for their own research questions. Finally, the EO signal in this paper is acquired with a commercial eye tracker (Tobii Pro Spectrum) and even though it has been validated internally by the manufacturer, we do not know how accurately it estimates true eye openness.

Regarding the detection of PS blinks, one possible improvement would be to use the artifacts around blinks in the PS signal that typically occur before and after blinks due to the gradual disappearance and re-appearance of the pupil in the eye image. While this has been used with EyeLink data (Mathôt, 2013), where this artefact seems to occur systematically, it is less clear whether it can be successfully applied to data for other eye trackers; we have seen several instances in the data from the Tobii Pro Spectrum where these characteristic artifacts are not present in the signal (cf. data in GitHub repo). Moreover, some authors explicitly exclude values in the pupil size signal that are not physiologically plausible, e.g., those where the reported pupil size is smaller than 0 (Kret & Sjak-Shie, 2019) or 2 mm (Alnæs et al., 2014), or values considered to be statistical outliers in the pupil size signal (Alnæs et al., 2014; Brych et al., 2021). Even though we have not used these exclusion criteria for PS blink detection in this paper, the option to use them is included in our algorithm publicly available on GitHub, such that readers can test the influence of the exclusion criteria on their own data sets. Importantly, neither using such exclusion criteria nor using a different algorithm for PS blink detection affect the results in a way that changes the conclusions of our paper (cf. the Appendix).

Finally, given the recent developments in computer vision and machine learning and the availability of open software such as OpenFace (Baltrusaitis et al., 2018) or MediaPipe (Lugaresi et al., 2019), eye openness can be estimated from the eye landmark positions these software packages provide. Consequently, our proposed blink detection algorithm could be used on eye-openness signals captured with standard web cameras. This would allow researchers to study blinks both outside of an eye-tracking context and co-study blinks and gaze behavior for those who already have a P-CR eye tracker.

### Conclusions

Using the eye-openness signal, our proposed algorithm provides more robust blink detection and more detailed information about blink parameters compared to blinks detected from the pupil-size signal. Therefore, we recommend users of an eye tracker providing an eye-openness signal to consider using this instead of the pupil-size signal.

**Fig. 8:**
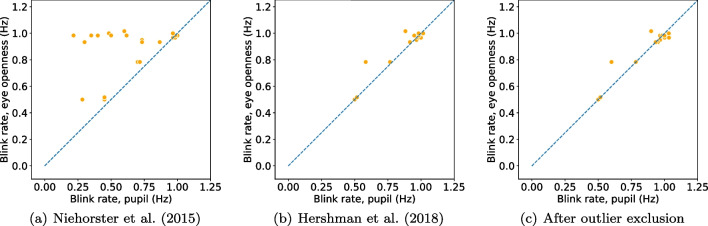
Comparison of blink rate detected from the eye openness and pupil size signals with three different algorithms for PS blink detection when participants are located in the center of the head box. These figures should be compared with Fig. 4a

**Fig. 9:**
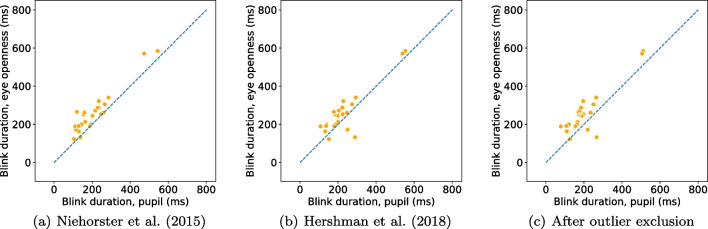
Comparison of blink duration detected from the eye openness and pupil size signals with three different algorithms for PS blink detection when participants are located in the center of the head box. These figures should be compared with Fig. 4b

**Fig. 10:**
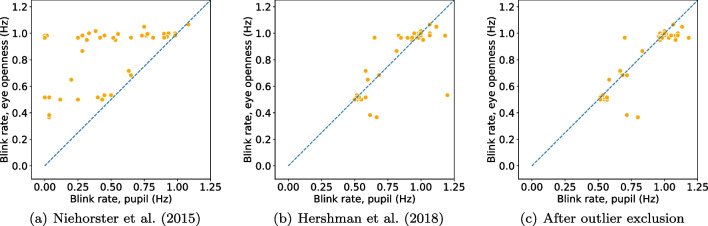
Comparison of blink rate detected from the eye openness and pupil size signals with three different algorithms for PS blink detection when participants are located at the edge of the head box. These figures should be compared with Fig. 5a

**Fig. 11:**
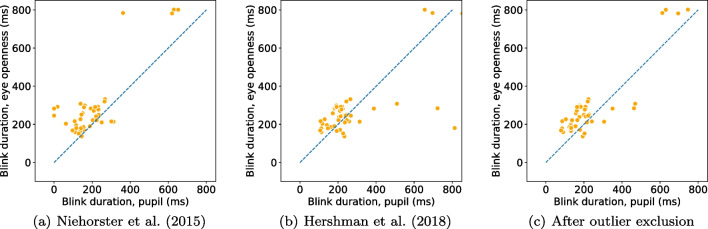
Comparison of blink duration detected from the eye openness and pupil size signals with three different algorithms for PS blink detection when participants are located at the edge of the head box. These figures should be compared with Fig. 5(b)

We hope that our publicly available blink detection algorithm together with the comprehensive review of the definition, operationalization, and classification of blinks will advance our knowledge of blinks in the future.

## An influence of algorithm and exclusion criteria on PS blink rate and duration

To investigate how the choice of algorithm and criteria for the exclusion of outliers and physiologically implausible values in the PS signal influence blink rate and duration, we tested two additional algorithms for PS blink detection developed by Hershman et al. (2018) and Niehorster et al. (2015)^2^ as well as added two exclusion criteria to our PS blink detection algorithm (Alnæs et al., 2014).

The algorithm by Niehorster et al. (2015) consistently underestimated the rate of PS blinks (Figs. 8(a) and 10(a)). Their algorithm was developed on data from an EyeLink 1000 and relies heavily on the artifacts that this eye tracker produces around blinks (cf. Fig. 1). Eye balling data and detected blinks from Niehorster et al.’s algorithm shows that the underestimation of blinks originates from the fact that such artifacts are not always present around blinks in data recorded from the Tobii Pro Spectrum and blinks are therefore sometimes not detected.

In contrast, the algorithm by Hershman et al. (2018) provides results similar to the PS blinks we report in this article, both in terms of rate and duration and for participants who are sitting in the center or at the edge of the eye-tracker’s headbox (Figs. 8(b),  9(b),  10(b),  11(b)). Since Hershman et al.’s algorithm extends blinks before and after periods of missing data, the durations of blinks (center condition) detected with the Hershman algorithm are longer ($M = 230.3$ ms, $SD = 108.7$ ms) compared to the PS blinks reported in this paper ($M = 199.4$ ms, $SD = 105.2$ ms), but still shorter than the EO blinks ($M = 261.5$ ms, $SD = 112.9$ ms). Consequently, using the algorithm by Hershman shifts the data points in Figs. 4(b) and 5(b) slightly towards the unity line. Note, however, that Hershman et al.’s algorithm provided sensible output only after interpolating gaps of missing data in the pupil signal, similar to what we do for PS blink detection in this paper.

The effect of removing pupil size values considered to be physiologically implausible (pupil size values smaller than 2 mm) or outliers (pupil size values more than 2.5 SD away from the trial mean) (Alnæs et al., 2014) on blink rate and duration can be see in Figs. 8(c),  9(c), 10(c), and 11(c). The results are almost identical to running the algorithm without any exclusion of data (compare with Figs. 4 and 5); differences in blink rate across any condition are smaller than 0.02 Hz and differences in blink durations are smaller than 6 ms.

## Footnotes

1 https://developer.tobiipro.com/commonconcepts/eyeopenness.html, retrieved Aug 21, 2023

## Declarations

### Open practices statement

Data and code are available at https://github.com/marcus-nystrom/BlinkDetector. None of the experiments were pre-registered.
